# Novel, cold-adapted D-laminaribiose- and D-glucose-releasing GH16 endo-β-1,3-glucanase from *Hymenobacter siberiensis* PAMC 29290, a psychrotolerant bacterium from Arctic marine sediment

**DOI:** 10.3389/fmicb.2024.1470106

**Published:** 2024-10-02

**Authors:** Do Young Kim, Yung Mi Lee, Jong Suk Lee, Chung-Wook Chung, Kwang-Hee Son

**Affiliations:** ^1^Microbiome Convergence Research Center, Korea Research Institute of Bioscience and Biotechnology, Daejeon, Republic of Korea; ^2^Division of Life Sciences, Korea Polar Research Institute, Incheon, Republic of Korea; ^3^Department of Bioindustry, Gyeonggido Business and Science Accelerator, Suwon, Republic of Korea; ^4^Department of Biological Sciences, Andong National University, Andong, Republic of Korea

**Keywords:** GH16, endo-β-1, 3-glucanase, cold-adapted enzyme, Arctic, marine sediment, psychrotolerant, *Hymenobacter siberiensis*

## Abstract

Endo-β-1,3-glucanase is a glycoside hydrolase (GH) that plays an essential role in the mineralization of β-glucan polysaccharides. In this study, the novel gene encoding an extracellular, non-modular GH16 endo-β-1,3-glucanase (GluH) from *Hymenobacter siberiensis* PAMC 29290 isolated from Arctic marine sediment was discovered through an *in silico* analysis of its whole genome sequence and subsequently overexpressed in *Escherichia coli* BL21. The 870-bp GluH gene encoded a protein featuring a single catalytic GH16 domain that shared over 61% sequence identity with uncharacterized endo-β-1,3-glucanases from diverse *Hymenobacter* species, as recorded in the National Center for Biotechnology Information database. The purified recombinant endo-β-1,3-glucanase (rGluH: 31.0 kDa) demonstrated peak activity on laminarin at pH 5.5 and 40°C, maintaining over 40% of its maximum endo-β-1,3-glucanase activity even at 25°C. rGluH preferentially hydrolyzed D-laminarioligosaccharides and β-1,3-linked polysaccharides, but did not degrade D-laminaribiose or structurally unrelated substrates, confirming its specificity as a true endo-β-1,3-glucanase without ancillary GH activities. The biodegradability of various substrate polymers by the enzyme was evaluated in the following sequence: laminarin > barley β-glucan > carboxymethyl-curdlan > curdlan > pachyman. Notably, the specific activity (253.1 U mg^–1^) and catalytic efficiency (*k*_*cat*_/*K*_*m*_: 105.72 mg^–1^ s^–1^ mL) of rGluH for laminarin closely matched its specific activity (250.2 U mg^–1^) and *k*_*cat*_/*K*_*m*_ value (104.88 mg^–1^ s^–1^ mL) toward barley β-glucan. However, the *k*_*cat*_/*K*_*m*_ value (9.86 mg^–1^ s^–1^ mL) of rGluH for insoluble curdlan was only about 9.3% of the value for laminarin, which correlates well with the observation that rGluH displayed weak binding affinity (< 40%) to the insoluble polymer. The biocatalytic hydrolysis of D-laminarioligosaccharides with a degree of polymerization between 3 and 6 and laminarin generally resulted in the formation of D-laminaribiose as the predominant product and D-glucose as the secondary product, with a ratio of approximately 4:1. These findings suggest that highly active rGluH is an acidic, cold-adapted D-laminaribiose- and D-glucose-releasing GH16 endo-β-1,3-glucanase, which can be exploited as a valuable biocatalyst for facilitating low temperature preservation of foods.

## 1 Introduction

β-1,3-Glucans, which are polysaccharides composed of D-glucose residues linked by β-1,3-glycosidic linkages in the main chain, are commonly found in nature as structural components of cell walls in filamentous fungi, yeasts, marine macroalgae, and plants (Barsanti et al., 2011). Furthermore, it has been shown that curdlan, a linear D-glucose-based β-1,3-linked polysaccharide, can be produced extracellularly by some bacterial species under specific culture conditions (Zhang and Edgar, 2014; Wu et al., 2016).

Endo-β-1,3-glucanases (EC 3.2.1.39), which are the principal enzymes involved in the breakdown of β-1,3-glucan polysaccharides, generally operate through the synergistic action mediated by exo- and endo-type β-1,3-glucanases (Linton, 2020; Jin et al., 2023). It has been reported that these endo-acting β-1,3-glucanases are associated with seven glycoside hydrolase (GH) families (16, 17, 50, 55, 64, 81, and 128) based on their structurally-related catalytic domains.^1^ However, the majority of known endo-β-1,3-glucanases are distributed among inverting GH enzymes from three families (55, 64, and 81) and the retaining GH enzymes that feature a β-jelly roll fold from family 16 (Shrestha et al., 2011). In the biotechnological context, these biocatalysts have attracted mounting interest in the food, agriculture, and pharmaceutical industries due to their anti-fungal properties against various fungal pathogens as promising biological control agents (Cheng et al., 2009; Woo et al., 2014). Additionally, they hold promise for the generation of fungal protoplasts (Gacto et al., 2000; Ferrer, 2006), β-1,3-glucan oligosaccharides with immune-activating properties (Vetvicka, 2011), and yeast extract (Ryan and Ward, 1985).

Similar to other biomass-deconstructing GH enzyme genes (Kim et al., 2016, 2018; Bai et al., 2021a; Yajit et al., 2024), the genes coding for a variety of unique endo-β-1,3-glucanases with distinct molecular and biocatalytic properties have been recently discovered from the whole genome sequences of mesophilic (Souza et al., 2019; Bai et al., 2021b; Calloni et al., 2023) and thermophilic (Chen et al., 2021) organisms. Metagenomic analyses have also proven the presence of a particular β-1,3-glucan-disintegrating system in the microbiota of hot spring (Burkhardt et al., 2019), compost (Feng et al., 2021), and insect guts (Warnecke et al., 2007; Scully et al., 2013). However, unlike various psychrophilic GH enzymes that exhibit different biocatalytic functions (Yang and Dang, 2011; Song et al., 2017; Kim et al., 2021, 2022), reports on cold-active or cold-adapted endo-β-1,3-glucanases from psychrotolerant or psychrophilic organisms in cold environments including polar regions are relatively scarce. To date, of such enzymes belonging to GH family 16, only two low temperature-active endo-β-1,3-glucanases from *Cryptopygus antarcticus* (Song et al., 2010) and *Pseudoalteromonas* sp. LA (Mitsuya et al., 2018) have been discovered and characterized at the molecular level.

Compared to mesophilic and thermophilic biocatalysts, psychrophilic enzymes are generally regarded as highly intriguing as potential candidates due to their lack of requirement for heat treatment processes that interfere with the cost-effectiveness, quality, and sustainability of bioindustrial production (Feller and Gerday, 2003; Santiago et al., 2016). Therefore, to explore cold-active or cold-adapted endo-β-1,3-glucanases with prominent biocatalytic activity at low temperatures (≤ 25°C), we conducted an *in silico* analysis of the whole genome sequences of some fibrolytic microorganisms isolated from environmental samples collected from the Arctic and Antarctic regions. Herein, we describe the genetic and enzymatic characteristics of a novel, low temperature-active, D-laminaribiose- and D-glucose-releasing GH16 endo-β-1,3-glucanase from *Hymenobacter siberiensis* PAMC 29290 that was isolated from surface sediment in the East Siberian Sea of the Arctic Ocean (Park et al., 2022).

## 2 Materials and methods

### 2.1 Chemical compounds

A series of D-laminarioligosaccharides [D-laminaribiose (L_2_), D-laminaritriose (L_3_), D-laminaritetraose (L_4_), D-laminaripentaose (L_5_), and D-laminarihexaose (L_6_)], curdlan [molecular weight (M.W.): 2,000 kDa] from *Alcaligenes faecalis*, carboxymethyl (CM)-curdlan (M.W.: 1,650 kDa), low viscosity barley β-glucan (M.W.: 179 kDa), and pachyman (M.W.: not indicated) were sourced from Megazyme International Ireland Ltd (Wicklow, Ireland). All other chemical compounds including D-glucose (L_1_), laminarin (M.W.: 5 kDa), sodium carboxymethylcellulose (CM-cellulose), locust bean gum, beechwood xylan, Avicel PH-101, oat spelts xylan, flake chitin from crab shells, and *para*-nitrophenyl (PNP)-sugar derivatives (PNP-xylopyranoside, PNP-glucopyranoside, PNP-mannopyranoside, and PNP-cellobioside) featuring a β-1,4-glycosidic bond were acquired from Sigma-Aldrich (St. Louis, MO, USA).

### 2.2 Cloning of the endo-β-1,3-glucanase gene

Preparation of genomic DNA from *H. siberiensis* PAMC 29290, which was cultured in R2A broth (BD Difco, Franklin Lakes, NJ, USA) for 14 d at 15°C, was performed using a Mini Tissue DNA kit (Cosmo Genetech Co. Ltd., Seoul, Korea). The resultant DNA was employed as a template for the polymerase chain reaction (PCR) to amplify the gene encoding recombinant mature endo-β-1,3-glucanase (rGluH) proteins. For this, two gene-specific primers containing the restriction sites NdeI and HindIII were synthesized as follows: GluH-F (5′-*CATATG*TGCACTGAAAAAGGCAGC-3′) and GluL-R (5′-*AAGCTT*TTACTTGTATTGCAGGTACTTCAC-3′). With a T100™ thermal cycler (Bio-Rad Laboratories, Inc., Seoul, Korea), the PCR reaction was conducted with a reaction mixture (50 μL) composed of 2.5 U of FastStart Taq DNA polymerase (Roche, Basel, Switzerland), 250 μM of each dNTP, 2 pmol of each primer, 20 ng of template DNA, and a PCR buffer. The template DNA was initially denatured at 95°C for 4 min, followed by 30 cycles of 30 s at 95°C, 30 s at 54°C, and 50 s at 72°C. Following PCR, the amplified products were separated by electrophoresis on a 1.2% agarose gel, and the target DNA fragments were subsequently extracted from the excised gel using a NucleoSpin Gel and PCR Clean-up (Macherey-Nagel, Düren, Germany). The isolated gene fragments (804-bp) were ligated into a pGEM-T easy vector (Promega, Madison, WI, USA) for 3 h at 16°C, and the resulting mixtures including pGEM-T easy/*gluH* vectors were transformed into *Escherichia coli* DH5α competent cells. To isolate the constructed vectors, shake-flask culture of the recombinant cells was carried out using 50 mL of ampicillin (100 mg/L)-containing Luria-Bertani (LB) broth (BD Difco, Franklin Lakes, NJ, USA) at 180 rpm for 12 h at 37°C. The recombinant plasmids were purified using a NucleoSpin Plasmid (Macherey-Nagel), after which they were digested with NdeI and HindIII to generate the *gluH* fragments with the corresponding cohesive ends. These DNA fragments, after another round of purification with a NucleoSpin Gel and PCR Clean-up (Macherey-Nagel), were cloned into a pET-28a(+) vector (Novagen, Darmstadt, Germany) with matched cohesive ends, followed by the introduction of the assembled pET-28a(+)/*gluH* vectors into *E. coli* BL21.

### 2.3 Production and purification of recombinant endo-β-1,3-glucanase proteins

For the production of rGluH proteins with an N-terminal (His)_6_-tag, the culture of recombinant *E. coli* BL21 cells harboring pET-28a(+)/*gluH* was performed in a rotary shaker (150 rpm) for 16 h at 28°C, employing a 5-L baffled flask including LB broth (1 L) and kanamycin (25 mg/L). At a point where the optical density of cells in liquid culture at 600 nm reached approximately 0.5, 1 mM isopropyl β-D-1-thiogalactopyranoside (IPTG) was introduced into the culture medium to induce the overproduction of rGluH. The rGluH-expressing cells were subsequently harvested from the culture broth by centrifugation (7,000 × *g*) for 20 min at 4°C and then frozen at -20°C for 3 h. Active (His)_6_-tagged rGluH proteins were liberated by ultrasonic disruption from the recombinant *E. coli* BL21 cells, which were thoroughly suspended in a binding buffer (pH 7.4) including 20 mM imidazole, 0.5 M NaCl, and 20 mM sodium phosphate. The soluble fraction, which exhibited laminarin-degrading activity, was then recovered by centrifugation (15,000 × *g*) for 20 min at 4°C. For the purification of rGluH proteins, the aforementioned soluble fraction was applied to a HisTrap HP (Cytiva, Uppsala, Sweden) (5.0 mL) column connected to a fast protein liquid chromatography system (Amersham Pharmacia Biotech, Uppsala, Sweden). Thereafter, the (His)_6_-tagged rGluH proteins were eluted from the column using a linear imidazole gradient (20–500 mM) at a flow rate of 2.0 mL/min, in accordance with the manufacturer’s guidelines. The fractions showing potent endo-β-1,3-glucanase activity against laminarin were collected, pooled, and desalted with a HiPrep 26/10 desalting column (Cytiva) employing 50 mM sodium phosphate buffer (pH 6.0) as the eluent. The active fractions were combined and maintained in ice water for subsequent analysis.

### 2.4 Analysis of proteins

Sodium dodecyl sulfate-polyacrylamide gel electrophoresis (SDS-PAGE) analysis was accomplished to determine the relative molecular mass (M*_*r*_*) of the denatured rGluH proteins in a 12.0% gel. Following electrophoresis, the separated protein bands were visualized by staining the gel with a 0.05% Coomassie Brilliant Blue R-250 solution (Bio-Rad Laboratories, Inc.) for 3 h. Protein quantitation was achieved using Bio-Rad Protein Assay Dye Reagent Concentrate (Bio-Rad Laboratories, Inc.) with bovine serum albumin as a standard.

### 2.5 Assay of endo-β-1,3-glucanase activity

Using 3,5-dinitrosalicylic acid (DNS) reagent and D-glucose as a standard, the endo-β-1,3-glucanase activity of rGluH was assayed by quantitatively determining the amount of reducing sugars released from the biocatalytic degradation of laminarin, as previously described (Kim et al., 2022). The standard assay mixture (0.5 mL) consisted of 1.0% laminarin and enzyme solution (0.05 mL) diluted in 50 mM sodium acetate buffer (pH 5.5). Enzyme assays were routinely conducted for 10 min at 40°C, after which the biocatalytic reactions were immediately terminated by adding 0.75 mL of the DNS reagent to the reaction mixture. The colorimetric analysis was performed by measuring the *A*_540_ value of its red-brown color developed by heating at 100°C for 5 min. One unit (U) of endo-β-1,3-glucanase activity was defined as the quantity of rGluH necessary to yield 1 μmol of reducing sugar per min under standard assay conditions.

### 2.6 Effects of pH, temperature, and chemical compounds on the endo-β-1,3-glucanase activity

The influence of pH on the endo-β-1,3-glucanase activity of rGluH for laminarin was examined by exposing samples to pH values ranging from 3.5 to 9.0 at 40°C for 10 min employing the following buffer systems at 50 mM: sodium acetate (pH 3.5–5.5), sodium phosphate (pH 5.5–7.5), and Tris-HCl (pH 7.5–9.0). The pH stability of rGluH in these pH buffers was investigated by assaying its residual biocatalytic activity after termination of the enzyme reaction conducted at 40°C for 10 min. Initially, the enzyme was incubated at the respective pH values in the absence of laminarin for 1 h at 3°C, and then the enzymatic degradation was started by adding the substrate to the reaction mixture. The optimal temperature of rGluH to deconstruct laminarin was determined by incubating it with the substrate at temperatures ranging from 15 to 55°C for 10 min in 50 mM sodium acetate buffer (pH 5.5). The thermostability of rGluH at temperatures of 15, 20, 25, 30, 35, 40, 45, 50, and 55°C was evaluated by ascertaining its remaining endo-β-1,3-glucanase activity after terminating the biocatalytic reaction performed with the enzyme that had been exposed to the corresponding temperature for 1 h in the absence of laminarin. In this case, the enzyme reaction was proceeded for 10 min at pH 5.5 after supplementing the substrate to the reaction mixture. The effects of divalent cations (each at 1 mM) and chemical reagents (each at 5 mM or 0.5%) on the endo-β-1,3-glucanase activity of rGluH were estimated after its preincubation for 10 min at 3°C in the assay mixture including 1% laminarin and the chemical of interest.

### 2.7 Analysis of the hydrolysis products

The biocatalytic degradation of laminarin (2 mg) and D-laminarioligosaccharides (L_2_–L_6_, each 1 mg) was conducted by reacting rGluH (2 μg) with the respective substrates in 50 mM sodium acetate buffer (pH 5.5) for 6 h at 35°C, during which the enzyme appeared fairly stable. Subsequently, the biocatalytic hydrolysis was completed by boiling the reaction mixtures for 5 min. The final hydrolysis products formed by the enzyme reactions were identified by liquid chromatography-mass spectrometry (LC-MS) employing L_1_ and a series of D-laminarioligosaccharides up to L_6_ as standards. Quantitative analysis of the hydrolysis products was performed using ultra high performance liquid chromatography (UHPLC) employing a Vanquish UHPLC system (Thermo Fisher Scientific Inc., Waltham, MA, USA) connected with an ACQUITY BEH Amide column (1.7 μm, 2.1 × 100 mm, Waters Corp., Milford, MA, USA) and Orbitrap Fusion (Thermo Electron Co., Waltham, MA, USA). The hydrolysis products were then eluted from the column employing a mobile phase composed of water with 0.1% NH_4_OH (solvent A) and acetonitrile with 0.1% NH_4_OH (solvent B), at a flow rate of 0.4 mL/min. The following solvent gradient was used: 85% solvent B at 0–3 min, 60% solvent B at 10 min, 40% solvent B 10.1–12 min. MS analysis was performed with negative ion mode and scan range m/z 140–1,400.

### 2.8 Binding assay

The substrate-binding affinity of rGluH proteins was assessed using diverse insoluble polysaccharides with distinctive microstructures including curdlan, pachyman, Avicel PH-101, oat spelts xylan, colloidal crab shell chitin, and ivory nut mannan. In order to prepare the polymeric materials free from water-soluble sugar molecules, they were repeatedly washed four times with distilled water, followed by a single wash with 50 mM sodium acetate buffer (pH 5.5). These prepared polymers were utilized in the investigation of rGluH-substrate binding as follows: After mixing the suitably diluted enzyme solution (6.0 U/mL) with an equal volume of insoluble polysaccharide in a 1.5 mL Eppendorf tube, the mixture was initially incubated on ice for 2 h being strongly stirred every 5 min. Next, the supernatant containing rGluH proteins unbound to the insoluble materials was carefully collected by centrifugation (12,000 × *g*) for 10 min at 4°C and applied directly to the measurement of protein concentration as well as residual endo-β-1,3-glucanase activity.

## 3 Results and discussion

### 3.1 Genetic characterization of the GH16 endo-β-1,3-glucanase gene

**FIGURE 1:**
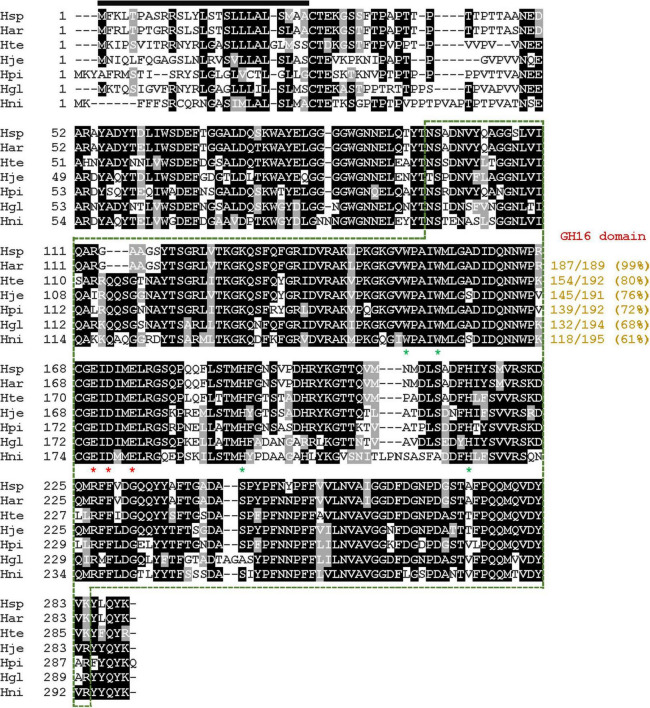
Alignment of the primary sequence of GH16 endo-β-1,3-glucanase from *Hymenobacter siberiensis* PAMC 29290 with those of other GH16 functional homologs. Shown are sequences (GenBank accession numbers) of *H. siberiensis* PAMC 29290 (Hsp) endo-β-1,3-glucanase (OQ589850), *Hymenobacter artigasi* (Har) GH16 protein (WP_168672369), *Hymenobacter terricola* (Hte) GH16 protein (WP_210513808), *Hymenobacter jejuensis* (Hje) GH16 protein (WP_139516443), *Hymenobacter piscis* (Hpi) GH16 protein (WP_215592205), *Hymenobacter glacialis* (Hgl) GH16 protein (WP_070735135), and *Hymenobacter nivis* (Hni) GH16 protein (WP_109655768). The identical and similar amino acids are displayed by black and gray boxes, respectively. The predicted signal peptide is indicated by a black bar and GH16 domain is outlined by a green dotted line. The catalytic triad (Glu170, Asp172, and Glu175) is indicated by red asterisks. Strictly conserved amino acid residues (Trp150, Trp154, His189, and His215) that take part in biocatalysis are marked with green asterisks.

It was predicted that the 870-bp GluH gene (GenBank accession number: OQ589850) identified through an *in silico* analysis of the whole genome sequence of *H. siberiensis* PAMC 29290 encodes an extracellular GH16 endo-β-1,3-glucanase composed of 289 amino acids (Figure 1). When examined using the Compute pI/MW tool,^2^ the premature GluH was evaluated to be a polypeptide having a deduced molecular mass of 31,934 Da and a calculated isoelectric point (pI) of 5.63, respectively. The signal sequence of the enzyme was predicted to be post-translationally modified between Ala26 and Cys27 in the N-terminal region, as assessed by the SignalP 6.0 server.^3^ However, in contrast to the premature GluH, its mature form without a signal sequence was characterized as an acidic protein having a deduced molecular mass of 29,110 Da and a calculated pI of 5.04, respectively. Meanwhile, the results of the protein BLAST and Pfam analyses suggested that similar to other uncharacterized GH16 functional homologs from *Hymenobacter* species deposited in the GenBank database (Figure 1), GluH might be a non-modular endo-β-1,3-glucanase comprised of a single catalytic GH16 domain (from Asn96 to Lys278). The domain structure of GluH closely resembled that of GH16 endo-β-1,3-glucanases from *Formosa algae* KMM 3553 (Kusaykin et al., 2017), *Pyrococcus furiosus* (Gueguen et al., 1997), *Fervidobacterium* sp. (Burkhardt et al., 2019), and *Cryptopygus antarcticus* (Song et al., 2010). On the other hand, several GH16 endo-β-1,3-glucanases were known to be modular multi-domain biocatalysts consisting of a catalytic GH16 domain along with an additional functional domain(s). For example, it has been shown that the domain architecture of an endo-β-1,3-glucanase (LamCAT) from *Aquimarina* sp. SCSIO21287 is composed of four distinct functional modules such as a catalytic GH16 domain, a crystalline domain, a carbohydrate-binding type 6 domain, and a Por secretion signal (Yang et al., 2020). Similarly, *Vibrio breoganii* 1C10 was a multi-domain endo-β-1,3-glucanase consisting of a catalytic GH16 domain, a carbohydrate-binding type 11 domain, and two carbohydrate-binding type 4 domains (Badur et al., 2020). Moreover, a tri-modular GH16 endo-β-1,3-glucanase (PglA) from *Paenibacillus* sp. S09 (Cheng et al., 2013) was made of an N-terminal leader region, a catalytic GH16 domain, and a C-terminal immunoglobulin-like domain. However, a bi-modular GH16 endo-β-1,3-glucanase (Actglu-CD) from compost was found to contain a carbohydrate-binding type 6 domain in its C-terminal region as an additional module (Feng et al., 2021).

**FIGURE 2:**
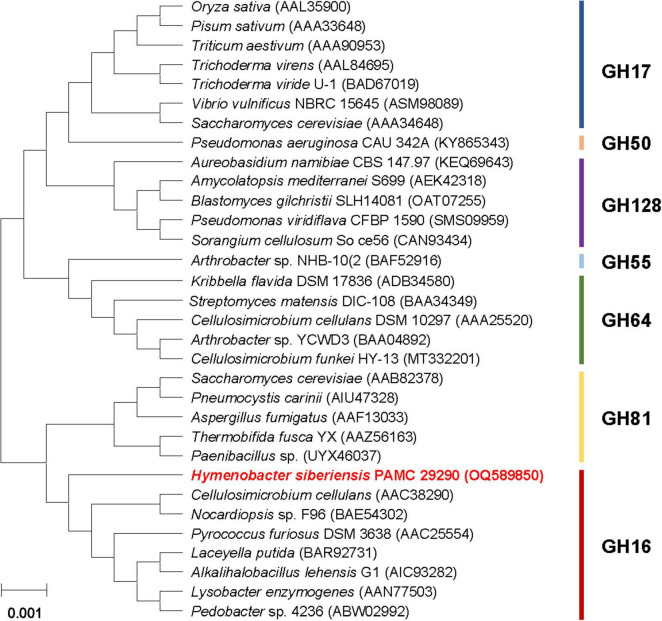
Phylogenetic analysis of *Hymenobacter siberiensis* PAMC 29290 GH16 endo-β-1,3-glucanase (GluH) and its functional analogs within seven GH families. Sequence alignment of the primary structures was performed using ClustalW in the MegAlign program (DNASTAR Inc., Madison, WI, USA). The primary sequence data employed for phylogenetic analysis were extracted from the GenBank database.

The phylogenetic analysis indicated that the primary sequence of premature GluH shared a close evolutionary relationship with that of the retaining GH16 endo-β-1,3-glucanases, as shown by alignment with its functional homologs within seven GH families (Figure 2). This result was solidly supported by multiple sequence alignment, which clearly revealed that the catalytic GH16 domain (from Asn96 to Lys278) of premature GluH from *H. siberiensis* PAMC 29290 shared high sequence identity over 61% with that of GH16 proteins from various *Hymenobacter* strains available in the National Center for Biotechnology Information (NCBI) database (Figure 1). However, these structural homologs of GluH have been molecularly and biocatalytically uncharacterized but just identified through a genome survey. Protein BLAST also indicated that the primary structure of GluH exhibited less than 56% sequence identity with known GH16 endo-β-1,3-glucanases from other eukaryotic and prokaryotic organisms belonging to different genera^4^ shown in Figure 2. Based on these results, this is the first report concerning the genetic and functional characteristics of a GH16 endo-β-1,3-glucanase from *Hymenobacter* species. The primary structure analysis of premature GluH revealed that its catalytic domain involved the two active site residues: Glu170 functioning as the nucleophile/base and Glu175 serving as the proton donor. These residues are strictly conserved among other GH16 endo-β-1,3-glucanases (Song et al., 2010; Mitsuya et al., 2018). Additionally, the two sugar binding sites (Trp150 and Trp154) in premature GluH, which are predicted to play a critical role in enzyme-substrate binding (Fibriansah et al., 2007; Song et al., 2010), were located in highly conserved regions of its catalytic GH16 domain.

### 3.2 Purification and electrophoretic analysis of rGluH

It has often been demonstrated that due to its relatively high hydrophobicity, certain GH enzymes such as endo-β-1,4-glucanase (Kim et al., 2016; Wierzbicka-Woś et al., 2019), endo-β-1,3-glucanase (Bai et al., 2021b), endo-β-1,4-xylanase (Kim et al., 2009, 2018), and chitinase (Wang and Wu, 2020; Bai et al., 2021a) in recombinant protein expression are produced primarily as inactive inclusion bodies. Similarly, the (His)_6_-tagged rGluH proteins were also produced as a mixture of active (< 20%) and inactive (> 80%) forms when overexpressed in pET-28a(+)/*gluH*-harboring *E. coli* BL21 under culture conditions. Thus, to examine the biocatalytic properties of rGluH, it was directly purified from a soluble cell lysate to electrophoretic homogeneity by basic Ni-NTA affinity column chromatography. The relative molecular mass of purified (His)_6_-tagged rGluH was determined to be 31.0 kDa by SDS-PAGE analysis, which was in good agreement with the calculated molecular mass (31,273 Da) of its primary structure estimated using the Compute pI/MW program.^5^ In terms of molecular mass, rGluH (31.0 kDa) consisting of a single catalytic GH16 domain was found to be similar to certain non-modular GH16 endo-β-1,3-glucanases such as *P. furiosus* LamA (30.0 kDa) (Gueguen et al., 1997), *C. antarcticus* CaLam (29.0 kDa) (Song et al., 2010), *Bacillus lehensis* G1 Bgl32 (31.6 kDa) (Jaafar et al., 2020), and *Fervidobacterium* sp. FLamA (30.0 kDa) (Burkhardt et al., 2019; Table 1). However, rGluH (31.0 kDa) was assessed to be smaller than two non-modular GH16 endo-β-1,3-glucanases [VbGH16A (53.0 kDa) and VbGH16B (41.0 kDa)] from *V. breoganii* 1C10 (Badur et al., 2020). Moreover, several multi-domain GH16 functional homologs with molecular masses ranging between 45.0 and 114.0 kDa were larger than the 31.0 kDa rGluH and have been identified in various prokaryotic and eukaryotic microorganisms (Hartl et al., 2011; Cheng et al., 2013; Mitsuya et al., 2018; Badur et al., 2020).

**TABLE 1:** Enzymatic characteristics of GH16 endo-β-1,3-glucanases.

Source	Enzyme	M*_*r*_* (kDa)	Opt. pH	Opt. temp. (°C)	Specific activity (U mg^–1^)	References
*Hymenobacter siberiensis* PAMC 29290	rGluH	31.0^a^	5.5	40	253.1^b^, 250.2^c^, 72.9^d^	This study
*Cryptopygus antarcticus*	CaLam	29.0	6.0	50	NI^e^	Song et al., 2010
*Pseudoalteromonas* sp. LA	LA-Lam	45.0	5.0	45	220.0^b^	Mitsuya et al., 2018
*Pyrococcus furiosus*	LamA	30.0	6.0	100	922.0^b^, 99.0^c^	Gueguen et al., 1997
*Bacillus lehensis* G1	Blg32	31.6	8.0	70	376.7^b^, 6.59^c^, 233.0^d^	Jaafar et al., 2020
*Fervidobacterium* sp.	FLamA	30.0	6.5	90	609.0^b^, 592.0^c^, 270.0^d^	Burkhardt et al., 2019
*Fervidobacterium* sp.	FLamB	30.0	6.5	90	876.0^b^, 648.0^c^, 434.0^d^	Burkhardt et al., 2019
*Paenibacillus* sp. S09	rPglA	91.0	5.5	60	2.01^b^, 0.08^c^	Cheng et al., 2013
*Vibrio breoganii* 1C10	VbGH16A	53.0	8.0	30	NI	Badur et al., 2020
*Vibrio breoganii* 1C10	VbGH16B	41.0	7.5	30	NI	Badur et al., 2020
*Vibrio breoganii* 1C10	VbGH16C	114.0	7.0	25	NI	Badur et al., 2020
*Aspergillus fumigatus*	rENG2	150.0	5.0–6.0	24–40	0.71^b^, 0.18^d^	Hartl et al., 2011
Compost	Actglu-CD	30.7	5.5	75	146.9^d^	Feng et al., 2021

^a^Calculated molecular mass.

^b^Specific enzyme activity toward laminarin.

^c^Specific enzyme activity toward barley β-glucan.

^d^Specific enzyme activity toward curdlan.

^e^Not indicated.

### 3.3 Enzymatic properties of rGluH

**FIGURE 3:**
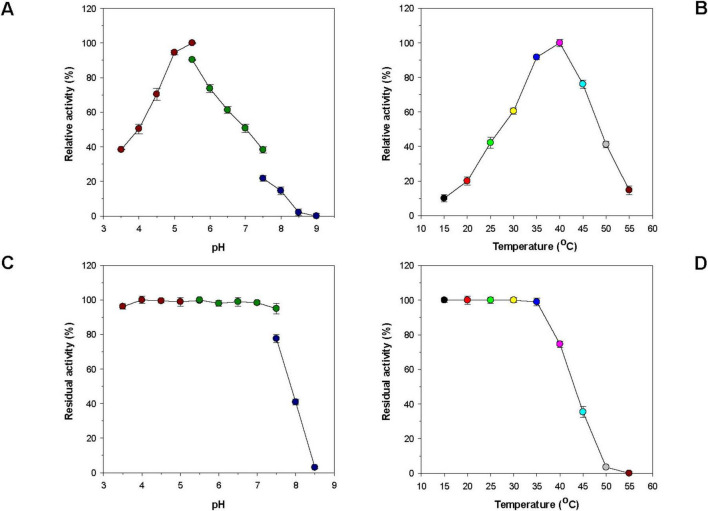
Effects of pH **(A)** and temperature **(B)** on the endo-β-1,3-glucanase activity of rGluH and effects of pH **(C)** and temperature **(D)** on the stability of rGluH. The optimum pH of rGluH was examined employing the following buffers at 50 mM: sodium acetate (pH 3.5–5.5), sodium phosphate (pH 5.5–7.5), and Tris-HCl (pH 7.5–9.0). The optimum temperature of rGluH was investigated at different temperatures (15–55°C) in 50 mM sodium acetate buffer (pH 5.5). The pH stability of rGluH was estimated by ascertaining the residual endo-β-1,3-glucanase activity after its pre-incubation using the aforementioned buffer systems (50 mM) at 3°C for 1 h. The thermostability of rGluH was evaluated by measuring the residual endo-β-1,3-glucanase activity after its pre-incubation at 15, 20, 25, 30, 35, 40, 45, 50, and 55°C in 50 mM sodium acetate buffer (pH 5.5) for 1 h. The values are mean ± SD of triplicate tests.

Unlike some alkaline GH16 endo-β-1,3-glucanases such as Blg32 (Jaafar et al., 2020), VbGH16A (Badur et al., 2020), and VbGH16B (Badur et al., 2020; Table 1), rGluH was an acidic endo-β-1,3-glucanase that showed the highest laminarin-degrading activity at pH 5.5 and 40°C (Figures 3A, B). The optimum pH of rGluH to degrade laminarin was similar to that of other microbial GH16 endo-β-1,3-glucanases, such as rPglA (Cheng et al., 2013), rENG2 (Hartl et al., 2011), and Actglu-CD (Feng et al., 2021), toward β-1,3-glucan substrate(s) (Table 1). However, its optimum pH (5.5) to hydrolyze laminarin was lower than that (6.5) found in two thermophilic endo-β-1,3-glucanases (FLamA and FLamB) from *Fervidobacterium* sp. (Burkhardt et al., 2019). It should be also noted that rGluH maintained stability at acidic and neutral pHs but it was drastically inactivated at alkaline pH values above 7.5 in a pre-incubation period of 1 h (Figure 3C). The pH stability of the enzyme was very comparable to that of an acidic endo-β-1,3-glucanase (rPglA) from *Paenibacillus* sp. S09, which retained over 80% of its original endo-β-1,3-glucanase activity even when subjected to pH 10.0 for 4 h (Cheng et al., 2013). It is also interesting to note that rGluH not only maximally catalyzed the hydrolytic degradation of laminarin at 40°C but also retained more than 40% of its original endo-β-1,3-glucanase activity even at 25°C for the same polysaccharide (Figure 3B). In addition, the enzyme showed approximately 12% of its maximum degradation activity for laminarin even at 15°C. However, rGluH was considered to be thermolabile as its biocatalytic activity and thermostability were significantly decreased in a temperature-dependent manner when exposed to temperatures exceeding 40°C (Figures 3B, D). Taken together, it was strongly suggested that similar to *Pseudoalteromonas* sp. LA GH16 LA-Lam (Mitsuya et al., 2018) and *C. antarcticus* GH16 CaLam (Song et al., 2010), rGluH was a new cold-adapted GH16 endo-β-1,3-glucanase active at low temperatures below 25°C. It is believed that the cold adaptation of rGluH might be attributed to an increase of its structural flexibility being induced at low temperatures, which enhances the substrate accessibility of enzyme (Parvizpour et al., 2015). As displayed in Table 1, the optimal temperature (40°C) of rGluH was assessed to be higher than that of three GH16 endo-β-1,3-glucanases [VbGH16A (30°C), VbGH16B (30°C), and VbGH16C (25°C)] from the marine bacterium *V. breoganii* 1C10 (Badur et al., 2020). Conversely, it has been reported that LamA (Gueguen et al., 1997), Blg32 (Jaafar et al., 2020), FLamA (Burkhardt et al., 2019), FLamB (Burkhardt et al., 2019), and Actglu-CD (Feng et al., 2021) are all thermophilic GH16 endo-β-1,3-glucanases, each showing maximal degradation of β-glucan(s) at substantially higher temperatures (100, 70, 90, 90, and 75°C, respectively), as seen in Table 1.

**FIGURE 4:**
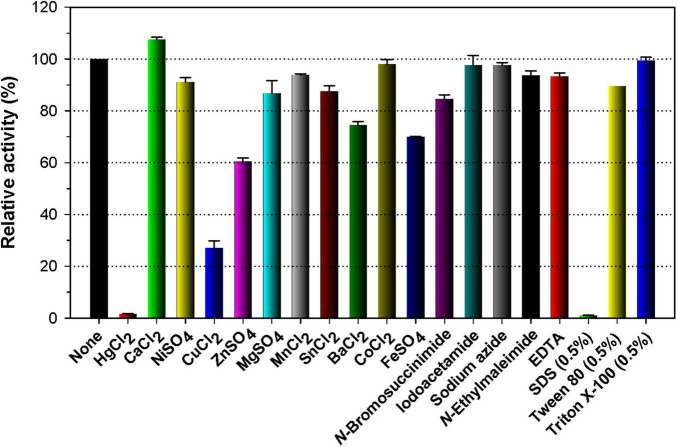
Effects of metal ions (1 mM) and chemical reagents (5 mM) on the endo-β-1,3-glucanase activity of rGluH. The values are mean ± SD of triplicate tests.

Like many other GH enzymes including endo-β-1,3-glucanase (Shrestha et al., 2011; Bai et al., 2021b), endo-β-1,4-glucanase (Yin et al., 2010; Kim et al., 2022), and endo-β-1,4-xylanase (Kim et al., 2009, 2010), it appeared that GH16 rGluH was almost fully inactivated when preincubated with a tryptophan (Trp) residue-directed modifier, Hg^2+^ (1 mM) for 10 min in the absence of laminarin (Figure 4). The robust inhibition of rGluH exerted by Hg^2+^ corresponded well to the understanding that this reactive chemical modifies the indole ring of active site Trp residues of carbohydrolases that play a crucial role in enzyme-substrate interaction (Zolotnitsky et al., 2004). Conversely, rGluH was relatively insensitive to *N*-bromosuccinimide (5 mM), similar to the previously reported suppression of *Microbacterium trichothecenolyticum* HY-17 GH10 endo-β-1,4-xylanase by the same Trp residue-specific modifier (Kim et al., 2014). It is also interesting to note that the biocatalytic activity of rGluH to hydrolyze laminarin was partially downregulated by approximately 72 and 39%, respectively, in the presence of 1 mM Cu^2+^ and Zn^2+^ ions. The enzyme inhibition by Cu^2+^ or Zn^2+^ was comparable to that observed in two GH16 endo-β-1,3-glucanases (FLamA and FLamB) from *Fervidobacterium* sp., which were nearly completely inactivated by the same metal ions (Burkhardt et al., 2019). Moreover, it has been reported that unlike rGluH, *B. lehensis* G1 GH16 Blg32 is not only insensitive to Zn^2+^ ions but also slightly suppressed by Cu^2+^ ions with a 19% reduction in its endo-β-1,3-glucanase activity (Jaafar et al., 2020). In this study, the modulation in rGluH activity by divalent cations (1 mM) including Ni^2+^, Mg^2+^, Mn^2+^, Sn^2+^, and Co^2+^ ions was insignificant because its endo-β-1,3-glucanase activity was either slightly enhanced or reduced to < 15% in their presence (Figure 4). However, it has been noted that *B. lehensis* G1 GH16 Blg32 is greatly upregulated by approximately 1.6-fold in the presence of 5 mM Mn^2+^ ions (Jaafar et al., 2020). Additionally, no considerable alterations in the biocatalytic activity of rGluH were observed when reacted it with laminarin in the presence of EDTA, sulfhydryl reagents (iodoacetamide, *N*-ethylmaleimide, and sodium azide), or non-ionic surfactants (Tween 80 and Triton X-100). In particular, the stimulatory or inhibitory effect of EDTA on the biocatalytic activity of rGluH closely resembled that seen in *Fervidobacterium* sp. GH16 FLamB (Burkhardt et al., 2019) or *B. lehensis* G1 GH16 Blg32 (Jaafar et al., 2020) treated with the same metal chelator. Of the tested chemical compounds, SDS exhibited profound toxicity to rGluH because the chemical compound completely abolished the biocatalytic activity of rGluH (Figure 4). A similar observation was also made when some cold-adapted GH8 endo-β-1,4-glucanases were reacted with cellulosic substrates in the presence of the compound (Bhat et al., 2013; Kim et al., 2022). On the other hand, *B. lehensis* G1 GH16 Blg32 was reported to be nearly insensitive to SDS (Jaafar et al., 2020), although in the cases of two *Fervidobacterium* sp. GH16 endo-β-1,3-glucanases (FLamA and FLamB), their endo-β-1,3-glucanase activities could be partially downregulated by < 71.0% in the presence of the anionic surfactant (Burkhardt et al., 2019).

### 3.4 Substrate specificity, kinetic parameters, and hydrolytic properties of rGluH

The substrate specificity of a cold-adapted GH16 rGluH was examined using di- and mono-saccharides substituted with PNP as well as diverse hexose- and pentose-based polysaccharides characterized by unique microstructures. As shown in Table 2, rGluH was found to efficiently deconstruct different types of β-glucan polysaccharides in the order of laminarin (5 kDa) > barley β-glucan (179 kDa) > CM-curdlan (1,650 kDa) > curdlan (2,000 kDa) > pachyman. The specific activities of rGluH toward laminarin and barley β-glucan were measured at 253.1 and 250.2 U mg^–1^, respectively, significantly exceeding by more than 2.8-fold its activity (88.8 U mg^–1^) toward CM-curdlan. Moreover, it is worth noting that the biocatalytic activity (88.8 U mg^–1^) of rGluH to degrade CM-curdlan was approximately 1.2-fold higher than its curdlan-degrading activity (72.9 U mg^–1^), indicating that the enzyme was more active on soluble β-1,3-glucan compared to insoluble β-1,3-glucan. On the other hand, it appeared that compared to the aforementioned β-1,3-linked polymeric materials, rGluH did not display any detectable hydrolytic activity against various β-1,4-linked PNP-sugar derivatives and polysaccharides including CM-cellulose, locust bean gum, and beechwood xylan. Taken together, it was strongly suggested that rGluH was a novel, true GH16 endo-acting β-1,3-glucanase with specificity only for β-1,3-linked polysaccharides, similar to other functional GH16 homologs (Gueguen et al., 1997; Song et al., 2010; Cheng et al., 2013; Jaafar et al., 2020). Moreover, it was evaluated that the substrate preference of rGluH was notably affected by the molecular weight and water-solubility of β-glucan polysaccharides. For example, the enzyme exhibited significantly higher biocatalytic activity toward water-soluble low molecular weight laminarin and barley β-glucan than water-insoluble high molecular weight curdlan. The laminarin-hydrolyzing activity (253.1 U mg^–1^) of cold-adapted rGluH was found to be approximately 1. 2-, 125. 9-, and 356.5-fold higher than the respective activities of a cold-adapted GH16 endo-β-1,3-glucanase (LA-Lam: 220.0 U mg^–1^) from *Pseudoalteromonas* species LA (Mitsuya et al., 2018), a modular GH16 endo-β-1,3-glucanase (PglA: 2.0 U mg^–1^) from *Paenibacillus* sp. S09 (Cheng et al., 2013), and a GH16 endo-β-1,3-glucanase (rENG2: 0.7 U mg^–1^) from *Aspergillus fumigatus* (Hartl et al., 2011; Table 2). Moreover, the biocatalytic activities of rGluH toward different β-glucans were very comparable to those of some bacterial GH16 endo-β-1,3-glucanases toward the same substrates. For instance, unlike rGluH, which readily degraded both laminarin and barley β-glucan comparably (Table 2), a GH16 endo-β-1,3-glucanase (Blg32) from *B. lehensis* G1 could efficiently decompose laminarin together with curdlan. However, its hydrolytic activity (approximately 6.6 U mg^–1^) for barley β-glucan was reported as less than 1.8% of its laminarin-degrading activity (376.7 U mg^–1^) (Jaafar et al., 2020). Likewise, the biocatalytic activity (99.0 U mg^–1^) of the hyperthermophilic *P. furiosus* GH16 LamA for barley β-glucan was measured at approximately 9.3% of its laminarin-hydrolyzing activity (922.0 U mg^–1^) (Gueguen et al., 1997).

**TABLE 2:** Hydrolysis activity of recombinant endo-β-1,3-glucanase (rGluH) toward different substrates.

Substrate	Main linkage type	Specific activity (U mg^–1^)^a^	Relative activity (%)
Laminarin	β-1,3 and β-1,6	253.1 ± 1.4	100.0
Curdlan	β-1,3	72.9 ± 1.6	28.8
CM-curdlan	β-1,3	88.8 ± 2.1	35.1
Pachyman	β-1,3	31.1 ± 3.2	12.3
Barley β-glucan	β-1,3 and β-1,4	250.2 ± 2.3	98.8
CM-cellulose	β-1,4	ND^b^	–
Locust bean gum	β-1,4	ND	–
Beechwood xylan	β-1,4	ND	–
PNP-glucopyranoside	β-1,4	ND	–
PNP-mannopyranoside	β-1,4	ND	–
PNP-xylopyranoside	β-1,4	ND	–
PNP-cellobioside	β-1,4	ND	–

^a^Specific activity was obtained from the three repeated experiments.

^b^Not detected.

Using non-linear regression of the Michaelis-Menten equation, the kinetic parameters of rGluH toward laminarin, curdlan, and barley β-glucan (each 0.2–1.2%) were determined under the optimal reaction conditions. As listed in Table 3, rGluH showed a *V*_*max*_ value of 480.89 U mg^–1^, a *K*_*m*_ value of 2.35 mg mL^–1^, and a turnover number (*k*_*cat*_) of 248.45 s^–1^ toward laminarin. These results suggested that the cold-adapted GH16 rGluH was significantly more active on laminarin than the cold-active GH16 CaLam from *C. antarcticus*, which had a *V*_*max*_ value of 32.20 U mg^–1^ and a *K*_*m*_ value of 9.98 mg mL^–1^ toward the same substrate (Song et al., 2010). In addition, the catalytic efficiency (*k*_*cat*_/*K*_*m*_: 105.72 mg^–1^ s^–1^ mL) of rGluH for laminarin was found to be approximately 2.8- and 27.9-fold greater than that (*k*_*cat*_/*K*_*m*_: 37.60 mg^–1^ s^–1^ mL) of thermophilic bi-modular GH16 Actglu-CD (Feng et al., 2021) and that (*k*_*cat*_/*K*_*m*_: 3.79 mg^–1^ s^–1^ mL) of tri-modular GH16 rPglA, respectively, for the same polysaccharide (Cheng et al., 2013). However, the *k*_*cat*_/*K*_*m*_ values (105.72 and 9.86 mg^–1^ s^–1^ mL) of rGluH toward laminarin and insoluble curdlan were lower than those (179.05 and 83.44 mg^–1^ s^–1^ mL) of alkaline GH16 Blg32 for these polysaccharides (Jaafar et al., 2020). Table 3 further reveals that rGluH, when reacted with barley β-glucan, presented a *V*_*max*_ value of 462.87 U mg^–1^, a *K*_*m*_ value of 2.28 mg mL^–1^, a *k*_*cat*_ value of 239.14 s^–1^, and a *k*_*cat*_/*K*_*m*_ value toward the substrate, indicating that the kinetic parameters of rGluH for barley β-glucan were comparable to those for laminarin.

**TABLE 3:** Kinetic parameters of recombinant endo-β-1,3-glucanase (rGluH) determined using different β-glucan polysaccharides.

Substrate	*V*_max_ (U mg^–1^)	*K*_m_ (mg mL^–1^)	*k*_cat_ (s^–1^)	*k*_cat_/*K*_m_ (mg^–1^ s^–1^ mL)
Laminarin	480.89	2.35	248.45	105.72
Curdlan	119.55	6.26	61.76	9.86
Barley β-glucan	462.87	2.28	239.14	104.88

**FIGURE 5:**
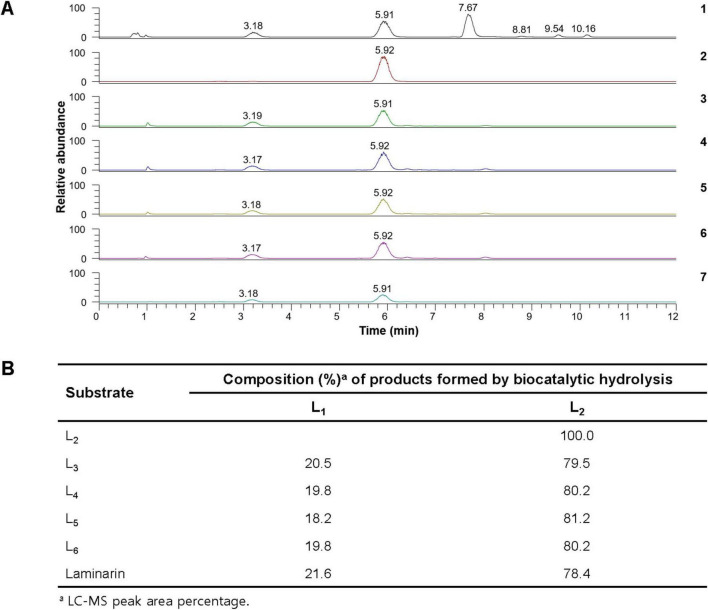
Liquid chromatography-mass spectrometry (LC-MS) analysis of the hydrolysis products of D-laminarioligosaccharides (L_2_-L_6_) and laminarin by rGluH **(A)** and composition of their hydrolysis products **(B)** (1) mass chromatogram of the standards [D-glucose (L_1_: a peak with a retention time of 3.18 min), D-laminaribiose (L_2_: a peak with a retention time of 5.91 min), D-laminaritriose (L_3_: a peak with a retention time of 7.67 min), D-laminaritetraose (L_4_: a peak with a retention time of 8.81 min), D-laminaripentaose (L_5_: a peak with a retention time of 9.54 min), and D-laminarihexaose (L_6_: a peak with a retention time of 10.16 min)]; (2) mass chromatogram of the hydrolysis products of L_2_; (3) mass chromatogram of the hydrolysis products of L_3_; (4) total ion chromatogram of the hydrolysis products of L_4_; (5) mass chromatogram of the hydrolysis products of L_5_; (6) mass chromatogram of the hydrolysis products of L_6_; (7) mass chromatogram of the hydrolysis products of laminarin.

The results of LC-MS analysis strongly indicated that rGluH could readily degrade laminarin and D-laminarioligosaccharides with a degree of polymerization in the range of 3–6 to release L_2_ as the predominant product and L_1_ as the minor product (Figures 5A, B). It is also noteworthy that the compositional ratio of L_2_ to L_1_ in the reaction mixtures was consistently evaluated to be approximately 4:1, regardless of the substrates used (Figure 5B). However, in these experiments, D-laminarioligosaccharides larger than L_2_ were not detected as the final hydrolysis products, implying that rGluH did not possess transglycosylation activity exhibited in some GH16 endo-β-1,3-glucanases (Sova et al., 2013; Badur et al., 2020). Additionally, in comparison with a GH16 endo-β-1,3-glucanase (Blg32) from *B. lehensis* G1, which showed hydrolytic activity against L_2_ (Jaafar et al., 2020), the inability of rGluH to cleave the same substrate indicated a lack of exo-enzyme activity. Therefore, based on the substrate specificity of rGluH and its degradation patterns of D-laminarioligosaccharides and laminarin, it was proposed that the enzyme was a typical endo-β-1,3-glucanase similar to other known GH16 functional homologs without transglycosylation and exo-β-1,3-glucanase activities (Song et al., 2010; Mitsuya et al., 2018). When compared to L_1_ and L_2_-releasing rGluH, several GH16 endo-β-1,3-glucanases have been reported to decompose laminarin to L_1_, L_2_, L_3_, and additionally L_4_ and/or L_5_. For example, it has been reported that two GH16 endo-β-1,3-glucanases (FLamA and FLamB) from the thermophilic *Fervidobacterium* sp. preferentially deconstruct laminarin to a mixture consisting of L_1_, L_2_, L_3_, and L_4_ (Burkhardt et al., 2019). Moreover, a GH16 endo-β-1,3-glucanase (Blg32) from *B. lehensis* G1 has been shown to degrade laminarin to a mixture of L_1_, L_2_, L_3_, and L_5_ (Jaafar et al., 2020). Furthermore, the breakdown of laminarin by a cold-active GH16 endo-β-1,3-glucanase (CaLam) from *C. antarcticus* has shown to yield a mixture of L_1_ to L_5_ as the degradation products (Song et al., 2010). Taken together, the biocatalytic characteristics and action mode of rGluH on laminarin confirmed in this study clearly demonstrated that it was a novel L_1_ and L_2_-releasing GH16 endo-β-1,3-glucanase different from other characterized GH16 β-1,3-glucan-deconstructing enzymes (Table 1).

### 3.5 Binding affinity of rGluH to water-insoluble polysaccharides

**FIGURE 6:**
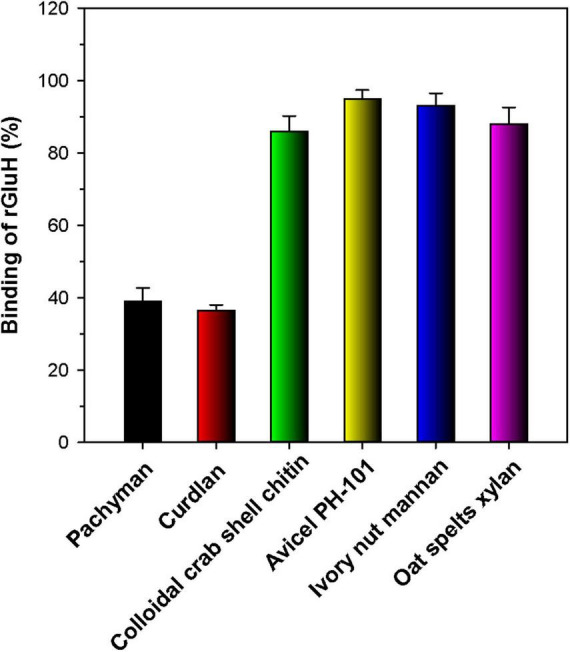
Binding of rGluH to water-insoluble polysaccharides. The values are mean ± SD of triplicate tests.

The substrate-binding ability of rGluH was evaluated by using various water-insoluble sugar-based polymers with distinctive microstructures (Figure 6). Unexpectedly, the results of the binding assays indicated that rGluH could tightly bind to structurally unrelated hydrophobic polymers such as Avicel PH-101, oat spelts xylan, ivory nut mannan, and colloidal crab shell chitin (Bai et al., 2021a), exhibiting high binding affinities (> 85%). Conversely, the enzyme displayed weak binding capacity to insoluble β-1,3-glucans such as curdlan and pachyman because its remaining endo-β-1,3-glucanase activity and protein concentration in the supernatant of reaction mixtures after termination of the binding experiments were assayed to be > 60%. The weak binding affinities of rGluH to curdlan and pachyman might be responsible for the observation that its endo-β-1,3-glucanase activities toward these insoluble substrates were substantially lower compared to those toward laminarin and barley β-glucan, which exhibit some degree of water solubility (Table 2). Thus, it is considered that due to the absence of an additional substrate-binding domain(s) in rGluH, its binding performance to the water-insoluble polymer powders with a low-surface area might be greatly reduced, similar to the findings in a bi-modular GH64 endo-β-1,3-glucanase from *Cellulosimicrobium funkei* HY-13 (Bai et al., 2021b).

## 4 Conclusion

The novel, acidic, cold-adapted β-1,3-glucan-disintegrating enzyme (GluH) from *H. siberiensis* PAMC 29290 is the first microbial GH16 endo-β-1,3-glucanase originated from the Arctic region that has been molecularly and enzymatically characterized. Compared to other known GH16 functional analogs (Table 1), the extracellular L_2_- and L_1_-releasing rGluH shows distinctive features in its amino acid sequence, thermal properties, substrate specificity, kinetic values for polymer substrates, and hydrolysis patterns of laminarin and D-laminarioligosaccharides (L_3_-L_6_). Due to its relatively high β-1,3-glucan-deconstructing activity even at 25°C (Figure 3B), the acidic, cold-adapted biocatalyst is expected to have great potential as a promising additive for the growth inhibition of pathogenic fungi in the low temperature processing industries of fruit and vegetable products, as described elsewhere (Qin et al., 2023). From a microbiological perspective, the current findings reflect the ecological significance of various β-1,3-glucan-degrading psychrophiles including *H. siberiensis* PAMC 29290, which participate in the biological recycling of food and agro-industrial by-products containing high β-1,3-glucan fractions in the polar region.

## Data Availability

The original contributions presented in this study are included in the article/Supplementary material, further inquiries can be directed to the corresponding author.

## Footnotes

1
http://www.cazy.org/glycoside-hydrolases.html

2
https://www.expasy.org/resources/compute-pi-mw

3
https://services.healthtech.dtu.dk/service.php?signalp-6.0

4
http://www.cazy.org/GH16_characterized.html

5
https://web.expasy.org/compute_pi/
