# Supplementary material for: Novel, cold-adapted D-laminaribiose- and D-glucose-releasing GH16 endo-β-1,3-glucanase from Hymenobacter siberiensis PAMC 29290, a psychrotolerant bacterium from Arctic marine sediment
Source: Front Microbiol. 2024 Oct 2;15:1470106. doi: 10.3389/fmicb.2024.1470106 (PMC11480075; doi:10.3389/fmicb.2024.1470106)
Supplement: Supplementary file 1 [file Data_Sheet_1.PDF]

GH family 16

>OQ589850 GH16 endo-β-1,3-glucanase [Hymenobacter siberiensis PAMC 29290]

MFKLTPASRRSLYLSTSLLLALSMAACTEKGSSFTPAPTTPTTPTTAANEDARAYADYTDLIWSDEFTGGALDQS
KWAYELGGGGWGNNELQTYTNSADNVYQAGGSLVIQARGAAGSYTSGRLVTKGKQSFQFGRIDVRAKLPKGKGVW
PAIWMLGADIDQNNWPRCGEIDIMELRGSQPQQFLSTMHFGNSVPDHRYKGTTQVMNMDLSADFHIYSMVRSKDQ
MRFFVDGQQYYAFTGADASPYPFNYPFFVVLNVAIGGDFDGNPDGSTAFPQQMQVDYVKYLQYK

>AAC38290.1 beta-1,3-glucanase II [Cellulosimicrobium cellulans]

MDLARHRSLTPPRTPTGRRPRARRRLASALVAALTAAAAALAVTVAATSAAAAPGDLLWSDEFDGAAGSAPNPAV
WNHETGAHGWGNAELQNYTASRANSALDGQGNLVITARREGDGSYTSARMTTQGKYQPQYGRIEARIQIPRGQGI
WPAFWMLGGSFPGTPWPSSGEIDIMENVGFEPHRVHGTVHGPGYSGGSGITGMYQHPQGWSFADTFHTFAVDWKP
GEITWFVDGQQFHRVTRASVGANAWVFDQPFFLILNVAVGGQWPGYPDGTTQLPQQMKVDYVRVYDNGSGSSSPG
NPGTGLPTGTGAVRAANGMCVDVPWADPTDGNPVQIVTCSGNAAQTWTRGSDGTVRALGKCLDVRDGSTTRGAAV
QVWTCNGTGAQKWAYDAGSKALRNPQSGLCLDATGGAPLRDGQRLQTWTCNGTTAQQWTL

>BAR92731.1 beta-1,3-glucanase [Laceyella putida]

MYTFGRLRRKGSIASRTFIVVMLILTVFGSVYTYPAGANASVPPTPPGWSLAFSDDFNGAQGSGVDRTKWIYDIG
HSYPGGAANWGTGEIEYMTDSTNNVYQDGSGHLVIKAIRDAGGGWTSGRIETQRTDFQPPAGGVMRVEASIQLPN
VTGAAAQGYWPAFWMLGAPFRGNYWNWPSIGEIDIMENVNGANTVWGTLHCGVNPGGPCNETSGIGGNRSGFSPS
LQTAYHTYAVEWDRSVSPEQIRWYVDGSLFHTVSANQVDATTWANATNHGFFIILNLAMGGGWPGNPTPSTASGA
SMNVDYVAVWTKQGGSGNPGDPGGSNYGVENVGSTQAKVWFKPPSTASYVILHYVQPGLSQQNVNMTYNSGAARW
EYTVSGLSSGQVLQYQFTYNIGGVQYDTAWYSYTKP

>AAN77503.1 beta-1,3-glucanase A [Lysobacter enzymogenes]

MKRLIEGLVLCLACAFAGSASAQSWQLVWSDEFNGSIGPSWVFETGNGSGGWGNNERQYYRRENAAIENNALVIT
ARRQDFNGFRYTSARMKTQGIRNFRYGKIEARMRLPSFMGAWPAFWALGANLPQVGWPDSGEIDIMEHINNENRS
YGTIHWRDHNGNYAQYGGNTAVSVADWHVYSVEWDANAIRWYVDGNKFHEANIQNNINGTEEFHRDFFLLLNFAI
GGNWPGFNIDESKLPAKMHVDYVRVYRRQ

>BAE54302.1 beta-1,3-glucanase [Nocardiopsis sp. F96]

MHRALFMAVVTAAALISPTTASASTTESDMRATLVWSDEFDGPAGSAPDPANWNHETGDHGWGNNELQNYTDSRA
NSALDGNGNLVITARQEADGGYTSARLTTQNKVQPQYGRVEASIQIPRGQGIWPAFWMLGADFPNTPWPDSGEID
IMENIGREPHLVHGSLHGPGYFGGEPLTGSYMHPQGWSFADTFHTFAVDWRPGSITWSVDGVAYQTYTSADTRGN
PWVFDQPFFMILNVAVGGDWPGYPDGSTQFPQEMRVDYVRVYELG

>ABW02992.1 glycoside hydrolase family 16 precursor [Pedobacter sp. 4236]

MKTINLMLCLIAGAQLLSSCKKDVNASSENSSTNTDPRAVSYQLLWSDDFDGNAVNQANWSFETGAGGWGNNEKQ
YYQPDNATVADGNLIITAKKQSVGGAPYTSARMITRGKKEYTYGRFEARIKLPQGQGQWPAFWMLGSNIGSVGWP
KCGEIDIMENVNTNTQVLGTIHWFDQAYAYYGGNTNTTPQNYHVYRVDWTPTSITWYVDNIQFHVANIANSINGT
DEFHRPFFLLLNMAVGGNLPGQTIDESRLPAKMYVDYVKVYKIVGS

>AIC93282.1 endo-beta-1,3-glucanase [Alkalihalobacillus lehensis G1]

MKKVIGALSIAACTTLFATSYAGAEETQGEPSIEQEGWNLVWSDEFDGNSLDPSKWRHDIGNGQPNLPGWGNEEL
QYYSDDPKNVRVENGELIIEAHQESVSDQYGTYGYTSGKVLTEGRFSQTYGRFEARMRLPAGQGFWPAFWMMPEN
DQYGGWAASGEIDIMENAGGTPNKVGGAIHYGGPWPENQFQAGDYYFPAGTDATGYHEYAVEWEPGEIRWYVDGN
HYQTINDWYSTGGSYPAPFDQDFHLILNLAVGGWYGGNPDGSTPFPSSMAVDYVRVYER

>AAC25554.2 endo-beta-1,3-glucanase precursor [Pyrococcus furiosus DSM 3638]

MKKEALLFLSLIFLVFVSGCIHHSTNQQLSSKQQVPEVIEIDGKQWRLIWHDEFEGSEVNKEYWTFEKGNGIAYG
IPGWGNGELEYYTENNTYIVNGTLVIEARKEIITDPNEGTFLYTSSRLKTEGKVEFSPPVVVEARIKLPKGKGLW
PAFWMLGSNIREVGWPNCGEIDIMEFLGHEPRTIHGTVHGPGYSGSKGITRAYTLPEGVPDFTEDFHVFGIVWYP
DKIKWYVDGTFYHEVTKEQVEAMGYEWVFDKPFYIILNLAVGGYWPGNPDATTPFPAKMVVDYVRVYSFVSG

GH family 17

>AAL35900.1 endo-1,3-beta-glucanase [Oryza sativa]

MARRQGVASMLTIALIIGAFASAPTTVQSIGVCYGVLGNNLPSRSEVVQLYKSKGINGMRIYYPDKEALNALRNS
GIALILDVGDQLSYLAASSSNAAAWVRDNVKPYYPAVNIKYIAVGNEVEGGATNSILPAIRNVNSALASSGLGAI
KASTAVKFDVISNSYPPSAGVFRDAYMKDIARYLATTGAPLLANVYPYFAYRGNPRDISLNYATFRPGTTVRDPN
NGLTYTNLFDTMVDAVYAALEKAGAGNVKVVVSESGWPSAGGFGASVDNARAYNQGLIDHVGRGTPKRPGPLEAY
IFAMFNENQKNGDPTERNFGLFYPNKSPVYPIRF

>AAA33648.1 beta-1,3-glucanase [Pisum sativum]

MASFFARTRRFSLVSLFLLELFTINLIPTTDAQIGICYGMMGNNLPPANEVIALYKANNIKRMRLYDPNQPALNA
LRDSGIELILGIPNSDLQTLATNQDSARQWVQRNVLNFYPSVKIKYIAVGNEVSPVGGSSWLAQYVLPATQNVYQ
AIRAQGLHDQIKVTTAIDMTLIGNSFPPSKGSFRSDVRSYLDPFIGYLVYAGAPLLVNVYPYFSHIGNPRDISLP
YALFTSPGVMVQDGPNGYQNLFDAMLDSVHAALDNTGIGWVNVVVSESGWPSDGGSATSYDNARIYLDNLIRHVG
KGTPRRPWATEAYLFAMFDENQKSPELEKHFGVFYPNKQKKYPFGFGGERRDGEIVEGDFNGTVSLKSDM

>AAA90953.1 beta 1,3-glucanase [Triticum aestivum]

MPLLILLMLLAAGAAGAESATPSLHIGVNYGANADNLPSPTSVATFLATKTTIDRVKLFDANPTFISAFAGTPIS
LAVSLPNSALPALADKATGLDAARSWIRANLSPYVPATNVTLLLAGNEILLSTDTNLILSLLPAMRRLAQALKAE
GLTGVRVTTPHYLGILAPSDGIPSNASFRAGYNTKLFPAMLQFHRDTGSPFMVNPYPYFSYRPETLNYALFRPNS
GIYDPATKLNYTSMLDAQMDAIYTAMKKLGYGDVDIAVGEAGWPTQAEPGQIGVGVQEARDFNEGMIRVCSSGKG
TPLMPNRTFETYLFSLFDENQKPGPIAERHFGLFNPDFTPVYDLGLLRDGASVAPTPSPNPSPNPSPKPAPSGGG
KWCVAKDGANGTDLQNNINYACGFVDCKPIQSGGACFSPNSLQAHASYVMNAYYQANGHTDLACDFKGTGIVTSS
DPSYGGCKYVS

>ASM98089.1 hypothetical protein AOT11_01225 [Vibrio vulnificus NBRC 15645 = ATCC 27562]

MNLMKNIIKSALLLGFSGLLVACGSDSKTEADSNTGTPNEVVLAPQPAPEGNYPTARNGEPLLGNANYPAISYGA
FRTTERTEANVPSVAELKEDLRLMEAMGIKVLRTYNTQGFSDTANLLIAIDELMAEDENFEMYVMLGIWIDALNS
WTSNPIDPTQNNPANYAEVAKAIEMVNAYPEIIKVLAVGNEAMVHWAPYHVTPTIILEHVNTLREKRDNGEIPAN
VWITSSDNFASWAGQGDYNHPDLAKLVEAVDYISLHSYPFHDTHYANAFWLVPEEEQSLTTIEKVDAAMLRAKQH
LLSQVKLVQDYLAGLGVNKQIHIGETGWASETNVMYGDEGSKAADEYKQKAFFDLMRAWSQEFGASLFFFQAFDE
PWKGAADNPGDSEKHFGLIDINGNAKYVIWDLVDAGAFNGITRAGLNIVKSQGGVEQNVLDSVLAPNAKPVVDQP

SDADQFIVLDSTLLNGGEAIAWEGTAYLAEESGVLTLTTPPTAGTAKDWGWGAGVVLAGQAGANLTGFENGSLSF
ELKGTTGSVLNIGFQTGLWGNNDRPQTNNFVLFGPTGRAISTEWTAYTIPMSELIKGNPDFSDVTSLIYFSGTAD
IDGGVVEVRNVVFNK

>AAA34648.1 exo-beta-1,3-glucanase [Saccharomyces cerevisiae]

MRFSTTLATAATALFFTASQVSAIGELAFNLGVKNNDGTCKSTSDYETELQALKSYTSTVKVYAASDCNTLQNLG
PAAEAEGFTIFVGVWPTDDSHYAAEKAALQTYLPKIKESTVAGFLVGSEALYRNDLTASQLSDKINDVRSVVADI
SDSDGKSYSGKQVGTVDSWNVLVAGYNSAVIEASDFVMANAFSYWQGQTMQNASYSFFDDIMQALQVIQSTKGST
DITFWVGETGWPTDGTNFESSYPSVDNAKQFWKEGICSMRAWGVNVIVFEAFDEDWKPNTSGTSDVEKHWGVFTS
SDNLKYSLDCDFS

GH family 50

>KY865343.1 beta-1,3-glucanase [Pseudomonas aeruginosa CAU 342A]

MIRSRWHLPLLLGLLAVATPLAASDIQQVLFNFVKPMAVVGITLEDADLPSATAEATPEGDILRRVTFSPAQRPT
LRMSPALGRWDWSAADYVSLRIQNAMSWDMTLEVAIEGEQGAPGLQASIELPAGPPQTLLVPLRAVSPEALGMRA
GPPMPQMVEGQRVLLAPRVEGSLDRARVGALSLSLRSPQAPQSILLGRFGIRTGRAVERSILTGLIDRYGQYSRA
DWPEKIRSDEQLRSAYAAEAAQLRDWERQTPARDRFGGLLGGPVFEASGFFRTEKRGGRWWLVTPEGHPFWSLGV
NAVTADGSRTYVEGREPMFAELPAEGEPLAAFFGEGDDRRGVAAQAGRRFGHGRWFDFLGANRQRIAPQASADQL
AGEWRRRTLERLSAWGFNSLGNWSDPALAAQARMPYSLPLSIAGDYATVSSGFDWWGAMPDPFDPRFAMAAERVI
AIAARDHRDDPWLLGYYADNELAWAGRDGSAQARYGLAFGALTLSMDSPAKRAFVKQLKAKYLGHEALAEAWGIE
LAAWEALEAPGYAAPLPGEGHPAIAEDYSAFLRLYADAYFKTLRDALQWHAPNHLLLGGRFAVSTPEAITSCARY
CDLLSFNLYTPLPGQGLDDSLLARLDKPVLISEFHFGSRDRGPFWGGVSEAANERARGDSYRTFLEAALKSPYIV
GAHWFQYLDQPASGRLLDGENGHIGLVGITGLPFAGFVDTVRRSNLAALSRLSAMARSMPAVEPLPPREDSAGS

GH family 55

>BAF52916.1 endo-beta-1,3-glucanase [Arthrobacter sp. NHB-10]

MTLSTTIPGVGRRARRARAVGAGVTALAAAATGVALAAPATAEPAPDPDLGPNVVFIDDSWSVDQINTYLASIND
EAEFSQDRHAVFFEPGVYGDASGEDDPATATGIVNAEVGYYTAIAGLGAQPDDVLINGALSVQPVVSCPNNPWSC
QDPGSLTRFWRSLSNVAVNPIQRPVDEDVTRPFPAGITDPHQLRFAVSQAAPLRRVDIQGDLTVFGRFGEYASGG
YLANSRVSGRLVTGSQQQWYTRNSEVGTWDGGVWNMVFSGVEGAPATDFGPKPDGTTGSKTTLDTTPLSREAPFL
FRDGEDLAVFVPNARTETRGVDWSTDADAGQKLSLDTFFVAHEGDSAAEINAALATGKNLLLTPGVYHLDEAIRV
ENPDTVVLGLGYASLAPTSGSAALEIGDVAGVKVAGITVDAGPVESDVLVRVGPADAHVADAADPTTLTDVFVRI
GGPWVGRATTSIEVNSDHVLMDHIWAWRGDHGAGIGWDQNTGDHGVVVNGDDVTATGLFVEHYQKNQTIWNGERG
RTVFYQSEIPYDPPSQAAFMDGDRKGFASYRVGDHVRRHDAVGLGVYSFFNQGVDVRVASGIQTPVRAGVTFRSM
TSVFLNGSGGIEHVVNDTGAPAVGSFASPQVVAYPTDLPAELDEQVRAGRRGPEEGRHHEDGGDEPLPDVPAQDL
LAGRRAAERADEQGAEQRQEDDRLDEPEEHGRTGRARRASARGRGRCRCRARTRRGRGAASVRRRVPGAGRRRWG

>AAL84695.1 beta-1,3-glucanase precursor [Trichoderma virens]

MLKLTAVVALLLGAASASPTPSPPASDEGITKRATSFYYPNMDHVNAPRGYAPDLDGNFNYQVYQTVNPGDGGAL
QRAITSDGSGSRHPQWFASQPRVVYIPPGTYTISQTLRFNTDTVLMGDPTNPPIIKAAAGFSGDQTLVSGQDPTT
NEKGELSFAVALKNVILDTTAIPGGNQFTALWWGVAQAAQLQNVKITMASSQNGNGHTGIRMGRGSTLGLADVRI

ERGQNGIWIDGHQQAAFHNIYFYQNTVGMLISGGNTFSIFSSTFDTCGTGISNTGGAPWIALIDAKSINSGVTFT
TTGMASFMIENLTKDNGTPVVVARGSTLVGASSHVNTYSYGNTVGRNPTYGDVTSSNTRPSGLAPGGRYPYVAPP
TYGDLPISSFLNVKDPAQNGGRTVKGDNTLDESGTLNAILELAASQNKVAYFPFGKYRVDSTLFIPKGSRIVGEA
WATITGNGNFFKNENSPQPVVSVGRPGDVGVAQIQDMRFTVSDALAGAIIVQFNMAGNNPGDVALWNSLVTVGGT
RGASALTNACGNPGNECKGAFIGIHLAKGSSAYIQNVWNWVADHIAESFSGGSSIAGKGGVLVESNGNKGTWLYA
LGSEHWWLYQLNLHNANNVVVSLLQSETNYEQGSNTQQIPPAPWVANVDTWGDPNFSWCNGGDKICRMGFGNYIN
GGSNIYTYASASWAFFSGPGYQGCSQFNCQQTMHWIAKTPSNLQAYGICSKDSVNTLRLGDGTFINTQNGYTGSW
SPGGGDVGRYTT

>BAD67019.1 laminarinase [Trichoderma viride U-1]

MLKSLAFAAALLGAVTASPTLARHEDRSLQPRANTSFWYAAMDHTGQYKGYAPHAPSPSSYNVFVAVNAGDAGSL
QSAIDSAGSSNRQNEWLASQPRVVYIPPGTYTLSQTLNMRTDTILMGDATNPPIIKAAAGFSGNYLVNGQDPSTG
VSGELSFAVGLKNLVLDTTAVSGTSSISALYWGVAQAAQLQNVKIVLAPSSGGKGHTGIQLGRGSTLGLADVRIE
NGQNGIWHNGHQQALYKSIYFYKNTIGMLISGGNTITLLNPTFDTCGTGISVTGGSPFVGIVDAKSINSGVTFTT
TVYPSIVIDNLSKDTSSDVVVLRGTTALKSSSKIVNYSYGNTVGRSPIFGAVSGTTARPAGVAPGGRIPAVVVPN
YAQNPVTDFVNVKDPSQNGGQTVKGDGSTDDSAALNKVLQFAAANNKIAYFPFGDYRVLSTLVVPVGSRLVGEAW
ATISGGGSFFKDASNPKPVVQVGNAGDVGVAQLQDFRFTVSDVLPGAIIVEFNAAGSNAGDVALFNSLITVGGTR
GADNLTNTCGKANAECQAAFLGLHFTKTSSAYVENTWNWVADHITEGFSGGSNIAAKGGALVESTKGTWLHGLGS
EHWWLYQLNLRSASNVVVSLLQSETNYDQGDNTQQVPPAPWKVDVNGWGDPDFSWCDSTARCHMGLANYVQGGSN
IYYFGSASWAFFSGPGYQGCASGGYQCQDYMHVIKTAPTNLQMYGMCAKDTSVALRLANGTNINAQPDFTGGWSP
GSDVGRYTT

GH family 64

>BAA04892.1 beta-1,3-glucanase [Arthrobacter sp. YCWD3]

MPHDRKNSSRRAWAALCAAVLAVSGALVGVAAPASAVPATIPLTITNDSGRGPIYLYVLGERDGVAGWADAGGTF
HPWPGGVGPVPVPAPDASIAGPGPGQSVTIRLPKLSGRVYYSYGQKMTFQIVLDGRLVQPAVQNDSDPNRNILFN
WTEYTLNDGGLWINSTQVDHWSAPYQVGVQRADGQVLSTGMLKPNGYEAFYTALESAGWGGLVQRAPDGSRLRAL
NPSHGIDVGKISSASIDSYVTEVWNSYRTRDMCVTPFSHEPGTQFRGRVDGDWFRFRNGSGQEVAAFKKPDASSV
YGCHKDLQAPNDHVVGPIARTLCAALVRTTALTNPNQPDANSAGFYQDARTNVYAKLAHQQMANGKAYAFAFDDV
GAHESLVHDGNPQAAYIKLDPFTGTATPIANGGSTEQPGTPGGLPAGTGALRIGSTLCLDVPWADPTDTNQVQLA
TCSGNAAQQWTRGTDGTVRALGKCLDVARSGTADGTAVWIYTCNGTGAQKWTYDSATKALRNPQSGKCLDAQGGA
PLRDGQKVQLWTCNQTEAQRWTL

>AAA25520.1 beta-1,3-glucanase [Cellulosimicrobium cellulans DSM 10297]

MPHDRKNSSRRAWAALCAAVLAVSGALVGVAAPASAVPATIPLTITNDSGRGPIYLYVLGERDGVAGWADAGGTF
HPWPGGVGPVPVPAPDASIAGPGPGQSVTIRLPKLSGRVYYSYGQKMTFQIVLDGRLVQPAVQNDSDPNRNILFN
WTEYTLNDGGLWINSTQVDHWSAPYQVGVQRADGQVLSTGMLKPNGYEAFYTALEGAGWGGLVQRAPDGSRLRAL
NPSHGIDVGKISSASIDSYVTEVWNSYRTRDMVVTPFSHEPGTQFRGRVDGDWFRFRSGSGQEVAAFKKPDASSV
YGCHKDLQAPNDHVVGPIARTLCAALVRTTALTNPNQPDANSAGFYQDARTNVYAKLAHQQMANGKAYAFAFDDV
GAHESLVHDGNPQAAYIKLDPFTGTATPLGNGGSTEQPGTPGGLPAGTGALRIGSTLCLDVPWADPTDTNQVQLA
TCSGNAAQQWTRGTDGTVRALGKCLDVARSGTADGTAVWIYTCNGTGAQKWTYDSATKALRNPQSGKCLDAQGGA
PLRDGQKVQLWTCNQTEAQRWTL

>ADB34580.1 Glucan endo-1,3-beta-D-glucosidase [Kribbella flavida DSM 17836]

MRIKPKLLAVLAAAATVAAGLTATVSAPAEAVPATIPLKITNNSGRGEPVYIYNLGTNLATGQQGWADANGTFHP

WPAGGNPPTPAPDASIAGPANGQSITLRMPKFSGRVYFSYGQKLVFKLTTGGLVQPAVQNPSDPNRNILFNWTEY
TLNDSGLWINSTQVDMFSAPYAVGVQRADGTTKVTGHLKPGGYNGFFTALRGQPGGWANLIQTAPNGTVLRALAP
SYGVEIGALPATVMDDYVNRVWSKYSTQTLTVTPFTDQPNIKYFGRVSGNVMNFTNSSGQVVTSFQKPNSASIFG
CAGLLDAPNDLVRGPISRTLCAGFNRSTLLTNPNQPDSSNVDFYKDVVTNHYSRKIHAQMADGKAYGFAFDDVGA
HESLVHDGNPQQAYITLDPFN

>BAA34349.1 laminaripentaose-producing beta-1,3-guluase (LPHase) [Streptomyces matensis DIC-108]

MLRTLRRRVTAVALGLATALGGGWLAAGVPSPAHAAVPATIPLTITNNSGRAEQIHIYNLGTELSSGRQGWADAS
GAFHPWPAGGNPPTPAPDASIPGPAPGRSTTIQIPKFSGRIYFSYGRKMEFRLTTGGLVQPAVQNPTDPNRDILF
NWSEYTLNDSGLWINSTQVDMFSAPYTVGVRRGDGTTLSTGKLRPGGYNGVFNALRGQSGGWANLIQTRSDGTVL
RALSPLYGVETGALPASVMDDYINRVWNKYTGTDLIVTPFADRPDVRYTGRVSGGVLRFTDGSGAVVTTFQKPDA
SSVFGCHRLLDAPNDQVRGPISRTLCAGFNRTTLLANPHQPDRSAAGFYQEPVTNHYARIIHAHMADGKAYGFAF
DDVGHHESLVHDGDPRGASLTLDPFD

>MT332201.1 beta-1,3-glucanase [Cellulosimicrobium funkei HY-13]

MSLDRRRRSRRAWAALCAAVLAVSGAVVGAAAPASAVPATIPLTITNDSGKGPIYLYVLGERDGVAGWADAGGTF
HPWPGGVGPVPVPAPDASIAGPGPGQSVTIRLPKLSGRVYYSYGQKMTFQIVLDGRLVQPAVQNDSDPNRNILFN
WTEYTLNDGGLWINSTQVDHWSAPYQVGVQRADGQVLSTGMLTPNGYEAFYTALESAGWGGLVQRAPDGSRLRAL
NPSHGIDVGKISSASIDSYVSEVWNSYRTRDMVVTPFSHEPGTQFRGRVDGDWFRFRNASGQEVAAFTKPDASSV
YGCHKDLQAPNDHVVGPIARTLCAALIRTTALTNPNQPDASDAGFYHDARTNVYAKLAHQQMANGRAYAFAFDDV
GAHESLVHDGNPQAAFIKLDPFTGAATPIGDDGGGTEQPNPGGGLPTGTGTIRAGAALCLDVPWADPTDTNQVQL
ATCSGNTAQQWTRGSDGTVRALGKCLDVARSGTADGTVVWIYTCNGTGAQQWVYDSGTQALRNPQSGKCLDAQGG
APLHDGQKVQLWTCNQTEAQRWSF

GH family 81

>AAB82378.1 Ylr144cp [Saccharomyces cerevisiae]

MCYSRQAIPPPVPNRPGGTTNRGPPPLPPRANVQPPVCSSENSSKPRENRVAGESLRTPSSSNPLADSQVNSDNI
FQSPVLSNLKAPPSVFNKVQHPVPKPNIDDQSVDPLETNKFYTNMLLDDNTQPIWTHPYSIWFSRDPELFGLAAN
HTLASQRVFDTTTNPPRFYFNPTNIKSFVFKAREFVSSNDIKLEFRDMKHMSMCLLMSLSSSQFIEFPLVQGMGF
VTAIYHDLGFELRSAVGFRSLERISVNERYGKYNIQLENNRNWILYLTSPDYSFPQDFQISLLDSNTIISSHKIN
GLICQLSADSVPSIDMAAGCYPVYCDLSGQTVDEHFTNYRFNYTVAGYSQSGTTLMYALPHHKAAFTPEMQEREI
ASSLDSTVKGLMTGYLTNSFDMQVQVPQELGFEPVALSLNKKADYSQEKLSKIREAAVQEVQLSDPQQESNIDSM
YFSGKILAKYAWILYVTHYILHDENLTKELLSKLTIAMERFISNQQVLPLNYDVSWKGIISSGSSSQDFGNSYYN
DHHFHYSYHVITAAIISLVDSDLSGVTNNSWLENNRDWVECLIRDYSGVDNDDPYFPQFRSFDWFNGHSWAKGLF
PSGDGKDEESTSEDVNSCYAIKLWGLVTGNSKLTDIANLQLGIMRNVFQSYFLYESNNTVQPKEFIGNKVSGILF
ENKIDHATYFGMEPQYIHMIHAIPITSASSWVRTPNFVKEEWEEKMQPIIDQVNDGWKGIIMLNMALLDPKFSYD
FFSQPDFNRNFLDNGQSLTWSLAYSGAFS

>AIU47328.1 endo-beta-1,3-glucanase [Pneumocystis carinii]

MRLLYGLFARRSGLHLCIIMVFFISYISSMPIMSYSNIYLTSTHLTPRFLGLPSFGFVFYIIDYIRELLRYLRGS
SKHDENIKPFNTTKIDVLTPITDRKPLDFFKPVNSTLSPLSIKDEDLKKPIQTNKFYSNLYLGNQRFPSFLDPYV
LTWTYGDYSGIAVAHSDDNQKVFEGGNPPKYFFNPLGVYSAVFSAEELKNANFTLTSLDQMSVNVIIKPNNSKGG
NLEMPLVRGMAYVTGIYTGLTPVFTSVVGFRSIEKEKKDDYYKFKATLHDEKKWLLYVFPKEMSEFDFIINGTTL
KATKGTFNGYIQVSKIPVDNDGAEGIIDASAGTYATKIILSASVSGNMGNYSFTFQTNDYKNRSLLHFAMPHHIA

SFDNDTTSRKTNFSLPSPTNGLMTAYTGKFWNMVENDLPTNISFSPYSPSGKEPSYSEEAKEAIKKAAQEEVAQN
FCSQLDPNSYYFSGKALSKFALLCYSIKTVLNNDTLFKECSKKLKDCLAPFVKNNYTYKLVYDQTWRGIVTERGF
IKEPFSDFGATFYNDHHFHYGYLIFAAAIMGYLDSEWIKENKDWCIDLMRDVANPVDDAYFPAFRYFDWFTGHSW
SKGLYESGDGKDEESSSEDYNFYFAAKLLGHSINDTVMISRSSLMLAILKRSLLSYFLYEPTNTIMPKSFIPNYV
AGIKFMNKIDHSTYFSPRLECIQGIHMLPLTSISPYIRIPSFVKSEWENKLQSIVGGIPDGWKGILYANLAISDP
KTSFNFFSKNFDKKFLDMGSSLTWYLVFSSAFLNSA

>AAZ56163.1 conserved hypothetical protein [Thermobifida fusca YX]

MSHASRRRWRRATTSAATAALLCGALLTFPSAPAAAQVRLGSGSYTTVLPPGASGPSDHTGAPVAPKVTADFTQP
VVTNDWWSSLIFQRYPGNPYGENLYAHPLSFKAQAHGLEVGYPDTPELVADGLKYQYTHSPDFVLGIHGLNAPAA
KVAGYSDWTVTADLSDGTRQLRTTIGQGLPFVYADVSGGPIRVEFTAPPTVWRRSGNAVGVTVNGHHYALFAPSG
TTWSESDTVFTADVGGSGYASVALLPSPDDFDRYAPYAYSFVTSTTLTYDYDPASATLTSTYRVTTEAREGTAQG
TLLALYPHQWKETTTALTDLSYASPRGPMRVVEGDRFTTELTTHGILPSLPTVDSADHQRLRALIDAELHASDPW
KGASDTYWTGKALGRLAQLVPIADSIGYTAGRDALLDLLKNKMEDWLTADGPGDNAQFYYDDQWDTLIGFPASFG
SNTELNDHDFHYGYFITAAATIARYDRSWISEERWGPMVTTVLRDANNPDRDDERFPWLRSFSPYAGHGWASGHA
GFASGNNQESSSEAMHFAASAALLGSLIGDEELRDLGVYLHTTQASAMRRYWQNADGDAFPAGYSHDVVGMVWSD
GGDHRIWWDGTPEELYGINYLPITAGSLYLGHDPEHAAAMHQSLVTRLGRQPQVWRDIHWAHQALSDPDAALAAF
EAQWQSYEPESGSSKAHTYQWLSTLAEFGTVDTSVTADTPHYAVFRDGDRRTYVAFNPTGQPLTVTFSDGTTLTV
PPGQLATG

>AAF13033.2 beta(1-3)endoglucanase [Aspergillus fumigatus]

MGIVSFLKPVVLANGQDVFVPVSTGPNSQDDWLEERSSVNTDTPVETNKFYCGLFLGTQTNNTFTHPYSVAWVKG
GGTSQSYGMAISHVESNIVAHGPVNTAIPGSPISYYVNPIGIHSVILSASELGPSTVLTTENPLPFSANAVLRPS
ASSSQSITIPVVQGMGFVTGIYSNLQPKIQSGVFFTKMVTAGSPRTGIFKYSLSLEDGTSWLLYATPDDGSDPQL
QLASNSEIIGPAGWSGTIQVAKNPAGASGEKFYDNSSGVYAVEGAVMGSVSESTGTYSLMWAKAGKDAQNTPLLM
FALPHHMESFDASTQSRATNITLRTTTKGQATAVIGEYWTMVEPELPISMGFAPWSVSGGSIDKISPAAQQVILA
AAPTELQQDMDAQTNLNSMYFSGKALSKFATLLYTVDKLGGNSTLAAEGLARLKQSFARFIDNRQQFPLVYDNVW
KGVVSSASYATGDVGADFGNTLYNDHHFHYGYFIHAAAIIGSMDPQWLETSKDWVNMLVRDAGNSAGNDPLFPFS
RGFDWFHGHSWAKGLFESFDGKDEESTSEDAMFAYALKMWGKTIGDVSMEARGNLMLGILRRSMRNYFLMESNNK
NHPANFIANKVTGILFENKVDHTTYFGNNLEYIQGIHMLPILPCSAFTRSKQFVKEEWDAMFASNGPDPAENVVG
GWKGVLYANLALVDPAASWNFFTQPNFDYSWIDGGASRTWYLAYAAGEFIID

>UYX46037.1 endo-beta-1,3-glucanase [Paenibacillus sp.]

MNKRIAWLLLLSLLAAVAVPAGAATAYSGEVALGAGSYSTVLPPGAVNVQSQIYKTGNVTGAMPTNDWWSNLAWD
TYSEAQYPHPLAMKNGSGGIRIYYPGNRITANSSCVCGWINDIHDFTVGHSAVASFPDAKVDGFSDWFVKAQYKS
GASEMNVSYGHGSPYVYFTYAGGSPKISFYDTPTIWSGSASTPVLGITVAGAHYGLFGASGTTWSGIGGKTLTNS
GTSYFSVAALPDNSAATLSKFAQYAYSHVTGTTASYSYNASASEVTTTYAFTTQAKQGTQTGTLFALYPHQWKNS
STALTSYTYNSVRGQMKVGEGSSFQTKMKYYGVLPSLPDKGSYNRQQLQQYVDQAEAETYTGDGDTYWIGKRLGK
LASLAPIADQVGDTTAANKFRSEIKTILQSWFKSSDSAGNLKSSQVFYYNNTWGTVIGYPASYGSNNELNDHHFH
YGYFIKAAAEIARVDKAWATQWGPMVNLLIRDIASSSRSDSMFPYLRNFDPYAGHSWAAGHARFGDGNNNESSSE
GMNAWAGMILWGQATGDTTARDTGIYLYTTEMNAINEYWFDVNNQNRPAGFTRSTASMVWGGKTVGDGTWWTGNP
EEVHGINWLPFTGASLYLTQYPDYTTRNYNALVSENGGTSFDAWEDLIYMYRAISNPGEAKSFWTSRGGALSAEA
GNSKAFAYHWIYNLDAIGNQDRTVTANTPAYAVFNKNGVKTYTAYNLTNSAITVTFSDGKTMSVPANGSATEGAG
GSNPTPTPTPTPTPTATPTATPTPTATPTPTPPAGQTYTHADFTATVTKSGSSESIAFTPSTAAAYVDVHYLVNG
ANQQNVRMIKSGSTWTHTIQGLTSGQSIELWFTYEKSGPQYDSPHYTYTH

GH family 128

>AEK42318.1 hypothetical protein RAM_19160 [Amycolatopsis mediterranei S699]

MNQFRKLLVLVTVAIAAIGGLAAPATAAGTKKGVSAAAFSGVTAALGDVGARWFYTWAADPQGITAPAGTEFVPM
IWGRDSVTADQLQRAKAAGSTLLAFNEPDLAGQANMSVETALDLWPQLQATGMRLGAPAVAYGGDTPGGWLDRFM
SGAAARGYRVDFIPLHWYGGDFSAAATGQLQSYLQAVYNRYHRPIWLTEYALTDFSGSTPRYPSAAEQADFVSRS
TAMLNGLSFVERYAWFSLSTSTTPTGLYTGTTPNSSGVAYRAAG

>SMS09959.1 hypothetical protein CFBP1590__2373 [Pseudomonas viridiflava CFBP 1590]

MILRKALQTRHALIAFALLLSVLSESAWTATKSVKRGVAYDVASPADLSALSTGMSWWYNWSPKPHDRLAAYDYA
GQYNVDFVPMVWNANLDDGQLKLYLLAHPGIRYLLVINEPNLVDQANMTPQAAAQLWPRLEQISAQTGVKLVGPA
MNWGTMTGYGDPVAWLDAFYAAYASAHQGRDPQIDYLAFHWYDYGLSSMLDRLSRYGKPFWVTEFANWHTLDDGL
QIDSLEKQKQQMAEMVTMLERRSDVFRYAWFTGRMTPDPHFSSLLDAEGRLTELGQYYLSLPYSE

>CAN93434.1 Hypothetical serine rich protein [Sorangium cellulosum So ce56]

MHIFRQPPARWLFIAAIASFAAVPACSSSDDEAQASSGSSGAGAGSASGSGAASSSGAGAGSSSSSSAASSSSAG
ASAGSASSSGAGSSSGSGGAQPTGCKRGLAYGYHSKADMDVLSPAVSWWYNWTHVPDEGVRPDYYRTLGVDYVPM
VWGGGNLDSAAAGRIASEIPEGARFLLGFNEPNFGAQADLSAAEAAALWPHVEAVADARGLALVSPAVNFCGGDC
QETDPFKYLDDFFAACSGCRVDYIGIHIYTGCKGEGDNQAQWLINHVETYKSRFDKPLWLTEFACDSAGSLAEQK
EFLVDALAYLENEPRIAKYAWFSGRADNVRHASLLGDDGELNELGQAYVSAPQHACGASTE

>KEQ69643.1 hypothetical protein M436DRAFT_66913 [Aureobasidium namibiae CBS 147.97]

MVKKRVLLWDYTNTRDVKWAMDKINFKGPLHSCSNWNTWYPDELKHRLPFRPMIHGKNNLTGGEWQNILKTNEEV
IHFFNEPERAGISPEEAAKIWNDQVLALRTSHHKRLVSPSCASDPAGIAWIKKWMNLVAKNPPDYLGLHWYGTKG
DEMIRYLESMHKEHPHQPIIVSEWASTSRSYPDVLGLTVQLANWMDSTPWVAEYALFGCMRQMADDFVSPEAQLM
NKDGSFTDLMWKYMSDQPMHI

>OAT07255.1 hypothetical protein BDBG_03339 [Blastomyces gilchristii SLH14081]

MVSFKSLLVTSALACAVSCAPAADYYTIAANPGSGKRGLAYNNINLLTAFEGGPFSWSYNWEPRPGGYTAGIEYV
PMLWGPRGYGSWNADAEAGIAAGSKNLLAFNEPDIASQANMSPEAAAAAYQKYMNPYAARARLGSPAVSNGAPPK
GLGWMQGFLDVCAGNCKIDFLAVHWHGPSGNVDDFKRYVSEAIALGQKYGIGTVWVTEFEGQGDEEAQVNFLKEV
LPWLDSNAGVERYASFFVDNLVKGGALTSVGKAYKTI
